# Brain structural changes after multi‐strategic metamemory training in older adults with subjective memory complaints: A randomized controlled trial

**DOI:** 10.1002/brb3.1278

**Published:** 2019-03-27

**Authors:** Jung‐Hae Youn, Seung‐Ho Ryu, Jun‐Young Lee, Soowon Park, Seong‐Jin Cho, Hunki Kwon, Jin‐Ju Yang, Jong‐Min Lee, Jiyeon Lee, Seolmin Kim, Gill Livingston, Dong Hyun Yoon

**Affiliations:** ^1^ Graduate School of Clinical Counseling Psychology CHA University Pocheon Republic of Korea; ^2^ Department of Psychiatry, School of Medicine Konkuk University, Konkuk University Medical Center Seoul Republic of Korea; ^3^ Department of Psychiatry Seoul National University & SMG‐SNU Boramae Medical Center Seoul Republic of Korea; ^4^ Department of Education Sejong University Seoul Republic of Korea; ^5^ Gachon University Gil Medical Center Incheon Republic of Korea; ^6^ Department of Biomedical Engineering Hanyang University Seoul Republic of Korea; ^7^ Department of Psychiatry Konkuk University Medical Center Seoul Republic of Korea; ^8^ Division of Psychiatry University College London London UK; ^9^ Institute of Sports Science Seoul National University Seoul Republic of Korea

**Keywords:** brain structure, cognitive function, metamemory, training

## Abstract

**Background:**

Metamemory is the process of monitoring and controlling one's memory. Improving metamemory may reduce the memory problem in old age. We hypothesized that metamemory training (MMT) would improve cognition in older adults with subjective memory complaints and change the brain region related to metacognition.

**Method:**

We recruited and randomized older adults to the multi‐strategic memory training of 10 weekly 90‐min sessions, based on the metamemory concept or usual care. Cognitive tests including the Elderly Verbal Learning Test, Simple Rey Figure Test, Digit Span, Spatial Span, Categorical Fluency, and the Boston Naming Test were done in 201 participants, together with magnetic resonance imaging (MRI) in 49 participants before and after training.

**Results:**

A total of 112 in the training group and 89 in the control group participated. The training group had a significant increase in long‐term delayed free recall, categorical fluency, and the Boston Naming test. In MRI, the mean diffusivity of the bundles of axon tracts passing from the frontal lobe to the posterior end of the lateral sulcus decreased in the training group.

**Conclusion:**

These results indicate that the MMT program has a positive impact on enhancing older people’ cognitive performance. Improved white matter integrity in the anterior and posterior cerebrum and increased cortical thickness of prefrontal regions, which related to metacognition, possibly suggest that the effects of the MMT would be induced via the enhancement of cognitive control.

## 1 INTRODUCTION

The increasingly aging population indicates that there is a growing need for maintaining and improving older peoples’ memory performance, which may possibly reduce the possibility of developing dementia. More than 50% of older people report subjective memory complaints (SMC) (Park et al., 2007), which constitute a cause of reduced self‐efficacy (McDougall & Kang, 2003). Individuals with subjective, but no objective, memory complains are twice as likely to develop dementia compared to those without SMC (Mitchell, Beaumont, Ferguson, Yadegarfar, & Stubbs, 2014), and the increased risk persists for decades (Kaup, Nettiksimmons, LeBlanc, & Yaffe, 2015). SMC may indicate that older people have a lack of metamemory ability, which is to understand and judge their own memory performance (Pannu & Kaszniak, 2005), although they may sometimes correctly identify an increased effort requirement in memory tasks. There is a strong correlation between metamemory accuracy and frontal lobe integrity (Lachman, 2006). Understanding how memory works and monitoring the memory processes can improve memory in old age through metacognitive training (e.g., (Kramarski & Mevarech, 2003)) this provides information on how our memory works and how we monitor and control the memory processes. Metamemory training (MMT) has had positive effects on everyday memory performance (McDougall & Kang, 2003) and has increased the executive functions related to metacognition. Brain imaging has been used to investigate the correlation between specific brain regions and improvement in cognitive ability (Bryck & Fisher, 2012; Green & Bavelier, 2008; Scholz, Klein, Behrens, & Johansen‐Berg, 2009). Cortical thickness, grey matter density, and white matter integrity in various regions of the brain can be enhanced by training, in older adults (Ballesteros, Kraft, Santana, & Tziraki, 2015; Cao et al., 2016). We have developed a multi‐strategic memory training based on the metamemory concept, which was efficacious in improving objective memory and fluency in a small controlled study with older adults (Youn, Lee, Kim, & Ryu, 2011).

Therefore, we hypothesized that this metamemory program could increase memory and executive cognitive performance in a larger sample of older adults with SMC and that scanning would demonstrate structural changes before and after the training. Since there have been no previous brain imaging studies on this subject, exploratory analyses based on whole‐brain imaging were performed.

## 2 MATERIAL AND METHODS

### 2.1 Ethical considerations

The study was conducted in accordance with the Helsinki Declaration and was approved by the Institutional Review Board of Seoul National University of Medicine.

### 2.2 Participants

The participants of the study were recruited from memory clinics and community‐based centers for dementia in Seoul, South Korea. They were recruited through a combination of web‐based, word‐of‐mouth, and community advertising. All participants reported SMC and expressed their wish to improve their memory ability.

#### 2.2.1 Diagnosis of SMC

The diagnosis of SMC took place through a questionnaire validated for the Korean population consisting of 14 items with dichotomous “yes” or “no” answers and a cut‐off value of > 5, as in previous studies (Youn et al., 2009). Four items measure subjective judgement of memory impairment and the other 10 items measure reported memory deficits in everyday life. Higher scores indicate higher perceived memory decline.

#### 2.2.2 Exclusion

Two geriatric psychiatrists screened the participants for dementia and other psychiatric disorders based on the criteria of the fourth edition of the Diagnostic and Statistical Manual of Mental Disorders (DSM‐IV) (American Psychiatric Association. & American Psychiatric Association. Task Force on DSM‐IV., 2000). Potential participants were excluded if they were less than 55 years old or met diagnostic criteria for dementia, had a history of alcohol or substance abuse, had experienced a head trauma with loss of consciousness lasting for more than 15 min, or if they had a severe medical illness, neurological or psychiatric disorders, other than dementia, visual or hearing difficulties that could interfere with the test taking procedure, or motor impairment that could affect the test scores.

### 2.3 Intervention

#### 2.3.1 Metamemory training program

The MMT program consists of an educational component and multi‐strategic training based on the metamemory concept for memory improvement. The metamemory concept educational component consists of the meta‐knowledge, meta‐monitoring, and meta‐judgment sections (Gilleen, David, & Greenwood, 2016). In the meta‐knowledge section, the participants obtain information on what they believe or think about their personal memory performance and understand how cognitive aging affects memory and how the brain operates in the process of memorization. Throughout this process, the older people are educated about efficient strategies for dealing with cognitive aging. In the meta‐monitoring and meta‐judgement sections, participants obtain the tools to judge their memory knowledge and performance. For example, during the learning of words, the participant must judge of his ability to recall each item by positioning on a frequency scale (from 0% to 100%). This prediction is compared to the effective memory performance. This comparison makes it possible to judgment of learning.

After the training, the participants are offered the practical opportunity to learn and apply the multi‐strategies in personal and group sessions. The program consists of 10 sessions at 1‐week intervals. Each session lasts 90 min and has one main theme based on the specific strategy designated to the session. The main themes are as follows: introduction of forgetfulness (session 1), memory process (sessions 2 & 3), memory structure (sessions 4 & 5), memory and attention (session 6), memory and brain (session 7), memory and environment (session 8), memory and perception (session 9), and memory and forgetting (session 10). The program used in this study is well described in a previous study (Youn et al., 2011).

#### 2.3.2 Assessment

1. We collected demographic information, including age, sex, and education.
2. Neuropsychological Measures

The Mini Mental State Examination (MMSE) is a neurocognitive test designed to screen cognitive impairment (Folstein, Folstein, & McHugh, 1975), with a score range from 0 to 30. Higher scores indicate better cognition. The Korean version of the MMSE has been validated for use with Korean older populations (Lee et al., 2008).

We used the Elderly Verbal Learning Test (EVLT) and the Simple Rey Figure Test (SRFT), as part of the Elderly Memory Disorder Scale, developed and standardized for the Korean older populations (Kim, Rim, Kim, & Lee, 2009), to test verbal and visual memory. In the EVLT, nine words from three categories are presented and the patient asked to immediately recall the learned word list five times. In addition, the long‐term (20 min) delayed free and cued recall and recognition tasks were administered. The results were scored from 0 to 9; higher scores indicate better verbal memory. In the SRFT, the copying and the drawing tasks on delayed recall after 20 min were included. The performances were scored from 0 to 16; higher scores indicate better visual memory.

To evaluate verbal and visual working memory, we used the Digit Span Test (DST) and the Spatial Span Test (SST). In the DST, participants were presented with a series of numbers and asked to repeat the list either forward or backward. In the SST, 10 cubes were located in a board and tapped in a certain sequence; the participants were told to mimic the sequence either forward or backward. The results were recorded for total scores of 0–14.

The Categorical Fluency Test (CFT) was used to test executive function and the short version of the Boston Naming Test (BNT) to examine language ability. In the CFT, participants were asked to name as many animals as possible within a minute. The number of valid responses was used as a score. In the BNT, 15 pictures were presented and participants were asked to name each presented stimulus. The number of valid responses measured in the BNT was recorded.

### 2.4 Brain imaging

#### 2.4.1 Diffusion‐weighted imaging data acquisition and processing

Diffusion‐weighted images were acquired using a 3.0 Tesla magnetic resonance imaging (MRI) scanner (Philips, Achieva, Philips Medical Systems, Best, the Netherlands). For Diffusion‐weighted imaging (DTI), a single‐shot twice‐refocused spin echo planar imaging pulse sequence with 32 diffusion sensitized gradient directions was utilized with the following imaging parameters: *b*‐value, 1,000 s/mm^2^; repetition time (TR), 7,259 ms; echo time (TE), 68 ms; flip angle, 90°; field of view (FOV), 220 mm; and matrix size, 128 × 128 pixels; slice thickness, 2 mm; and voxel size, 1.53 × 1.53 × 2 mm^3^.

The DTI data were processed using functional MRI of the Brain (FMRIB)’s Software Library (FSL) software (http://www.fmrib.ox.ac.uk/fsl). Motion artifacts and eddy current distortions were corrected by normalizing each diffusion weighted volume to the non‐diffusion weighted volume (*b*0), using the affine registration method in the FMRIB's Linear Image Registration Tool. Diffusion tensor matrices from the sets of diffusion‐weighted images were generated using a general linear fitting algorithm. Subsequently, fractional anisotropy (FA) and mean diffusivity (MD) were calculated for every voxel according to standard methods.

#### 2.4.2 MRI data acquisition and processing

T1 weighted MR images were acquired using a 3.0 Tesla MRI scanner (Philips, Achieva) with the following imaging parameters: repetition time (TR), 9.9 ms; echo time (TE), 4.6 ms; flip angle, 8°; FOV of 220 mm; and matrix size of 220 × 220 pixels; slice thickness, 1 mm; voxel size of 1 × 1 × 1 mm.

Structural MRI data were automatically processed with the CIVET pipeline to measure cortical thickness (Zijdenbos, Forghani, & Evans, 2002). A detailed image processing was described in Zijdensbos et al. (2002). In brief, the image processing included the following: correction for intensity nonuniformity (Sled, Zijdenbos, & Evans, 1998), normalization to the MNI 152 template (Collins, Neelin, Peters, & Evans, 1994), removal of non‐brain tissues (Smith, 2002), tissue classification of white matter, gray matter, cerebrospinal fluid, and background (Zijdenbos et al., 1996), and surface extraction of the inner and outer cortex (Kim et al., 2005; MacDonald, Kabani, Avis, & Evans, 2000). A surface model for each hemisphere consisted of 40,962 vertices. The surfaces were transformed back into the native space and cortical thickness was measured as the Euclidean distance between linked vertices of the inner and outer surfaces (Lerch & Evans, 2005). The cortical thicknesses was spatially registered onto a template surface (Lyttelton, Boucher, Robbins, & Evans, 2007; Robbins, Evans, Collins, & Whitesides, 2004) with a smoothing kernel of 20 mm (Lerch & Evans, 2005) to compare the thicknesses across participants.

#### 2.4.3 Procedures

Two hundred seventy‐five participants were randomly assigned to either the MMT condition (*n* = 150) or the control condition (*n* = 125). The randomization procedure was as follows: First, random digits according to the table of random numbers were generated. If the random digit was an even number, we assigned the participant to the control group; if it was an odd number, we assigned the participant to the training group. Neuropsychological measures were evaluated before the training (pre‐test evaluation) and after the training (post‐test evaluation). Two clinical neuropsychologists masked to randomization status conducted the neuropsychological assessments. Program participation was free of charge and there was no financial reward for participation. Those in the control condition received one session in which general education on memory, but no structured cognitive training was offered. Among the participants who reported that they were willing to undergo brain image scanning, we randomly selected 49 (39 from the training group, 10 from the control group) by following the odd (receive brain imaging) and even rule (not receive brain imaging). Magnetic resonance imaging was conducted immediately before the training (pre‐test evaluation) and within 4–8 weeks after the training (post‐test evaluation).

### 2.5 Statistical analysis

#### 2.5.1 Demographic and neuropsychological assessment

Statistical analyses of the demographic and neuropsychological assessment between groups were performed using PASW 18.0 (PASW, IBM, Somers, NY). Independent samples *t *tests were performed to compare age, educational level, and baseline neuropsychological scores. Chi‐square tests were used to analyze the sex ratio. Repeated measures analysis of variance (ANOVA) was conducted to examine the effect of the MMT on neuropsychological tests (i.e., EVLT immediate free recall, EVLT delayed free recall, SRFT copy, SRFT delayed recall, DST forward, DST backward, VST forward, VST backward, categorical fluency, Boston naming test). Analyses were performed with the training or control group as a between‐subject variable and the neuropsychological testing performance on the first and second follow‐up phases as the within‐subjects factor followed by Bonferroni correction for multiple comparison. When violations of sphericity were encountered, the Greenhouse‐Geisser correction was employed.

#### 2.5.2 Brain imaging analysis

The FA and the MD map of the DTI preprocessing results were used in the tract‐based spatial statistics (TBSS) analysis (Smith et al., 2006). All FA images were aligned onto the standard FMRIB58 FA template, included in the FSL software, using a nonlinear registration algorithm implemented in the TBSS package. The FA images, aligned on the FMRIB58 FA template, were averaged to create a skeletonized mean FA image. Each participant's aligned FA images were projected onto the skeleton by filling the skeleton with the highest FA values from the nearest relevant center of fiber tracts. A threshold FA value of 0.2 was chosen to exclude voxels of adjacent gray matter or cerebrospinal fluid. For the MD analysis, the MD images were also processed by applying the FA non‐linear registration and were projected onto the skeleton using projection methods identical to those inferred from the original FA data. Then, voxel‐wise statistics across participants on the skeleton‐space FA and MD images were performed.

A voxel‐wise statistical analysis of the individual skeleton images was performed using a nonparametric permutation test. Age and sex were included as covariates in the analysis of covariance and the null distribution was built up over 5,000 permutations. For control over multiple comparison correction, we used threshold‐free cluster‐enhancement with the “2D” parameter settings (Smith & Nichols, 2009). The results for FA and MD were considered significant for family‐wise error‐corrected *p* < 0.05.

To test the vertex‐wise group difference in cortical thickness of baseline (pre‐time point) and in cortical thickness changes (post‐time point–pre‐time point), we applied a general linear model using the SurfStat toolbox (http://www.math.mcgill.ca/keith/surfstat/) for Matlab (R2012a, The MathWorks, Inc., Natick, MA) with age, sex, and intracranial volume as covariates. For visual inspection and display purposes, the statistics results were mapped onto the MNI 152 brain‐surface models. The false discovery rate (FDR) correction for multiple comparisons (Genovese, Lazar, & Nichols, 2002) and an uncorrected *p* value of <0.001 were used.

## 3 RESULTS

**Figure 1:**
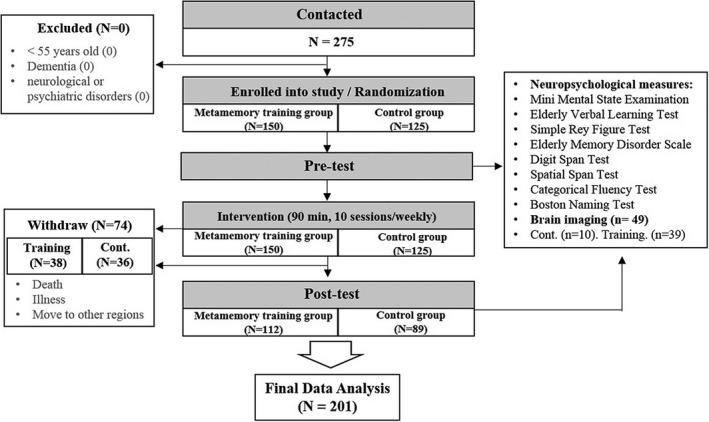
Summary of the trial progression of metamemory training

We recruited 275 people aged over 55 years with SMC from nine community centers (Figure 1) and were able to analyze 201 (73%) for cognitive outcome (112 from the training group, 89 from the control group). Seventy‐four participants (23%; 38 from the training group, 36 from the control group) dropped out due to death, illness, or move to other regions.

### 3.1 Demographical and neuropsychological state

Table 1 shows the demographic variables and the MMSE, Subjective Memory Complaints Questionnaire (SMCQ) scores. There were no significant differences between groups in demographic features or baseline MMSE and SMCQ score.

**Table 1:** Demographic variables and MMSE scores for each group

	Group		*t* or *x*	*p*	Total (*N* = 201)
	Training (*n* = 112)	Control (*n* = 89)			
Age (years)	69.93 (5.10)^a^	69.11 (4.6)	1.17	0.242	69.57 (4.90)
Education (years)	10.01 (3.89)	10.09 (3.52)	0.15	0.879	10.04 (3.72)
Gender (M:F)	48:64	29:60	2.21	0.147	77:124
MMSE	26.94 (2.6)	27.28 (2.2)	0.99	0.322	27.09 (2.44)
SMCQ	5.47 (3.34)	5.34 (3.23)	0.29	0.771	5.41 (3.28)

a *M*(*SD*), M: Male, F: Female, MMSE: Mini‐Mental State Examination.

### 3.2 Differences in neuropsychological tests and brain structure for each group

The repeated measures ANOVA revealed that there were significant group‐by‐time interactions in long‐term delayed free recall of verbal memory, categorical fluency, and the Boston naming test (Table 2). The simple effects analysis in each group indicated that significant increases in the long‐term delayed free recall of verbal memory (post‐pre mean difference (*SE*) = 0.90 (0.16), *t *(111) = 5.09, *p* < 0.001), categorical fluency (post‐pre mean difference (*SE*) = 1.63 (0.35), *t *(111) = 4.74, *p* < 0.001), and the Boston naming test (post‐pre mean difference (*SE*) = 0.54 (0.11), *t *(111) = 5.24, *p* < 0.001) were observed in the training group, while there were no significant changes on these three scores in the control group. These results showed the positive training effect in the training group compared to the control group.

**Table 2:** Comparisons between the training and control groups on memory performance

Measure	Training		Control		*F*(*df*)	*p* c	*η* ^2^
	Pre	Post	Pre	Post			
MMSE	26.94 (2.58)^a^	27.58 (2.05)	27.28 (2.25)	27.61 (2.23)	1.19	0.277	0.006
SMCQ	5.47 (3.34)	4.68 (2.93)	5.34 (3.23)	4.76 (3.40)	0.01	0.952	0.000
Verbal memory							
Immediate free recall^b^	29.71 (6.10)	31.96 (6.20)	29.58 (5.29)	32.07 (5.24)	0.12	0.731	0.001
Delayed free recall	5.44 (2.51)	6.34 (2.51)	5.91 (1.97)	6.24 (1.93)	6.00	0.015	0.029
Visuospatial Memory							
SRFT copy	15.10 (1.03)	15.13 (0.86)	15.14 (0.94)	15.15 (0.91)	0.01	0.914	0.000
SRFT delayed recall	11.34 (3.68)	12.57 (3.22)	12.06 (2.97)	12.78 (2.30)	1.97	0.162	0.010
Attention							
DST forward	5.53 (1.11)	5.70 (1.11)	5.65 (1.17)	5.91 (1.16)	0.35	0.555	0.002
DST backward	3.87 (1.08)	3.96 (1.08)	4.00 (1.19)	4.11 (1.11)	0.02	0.877	0.000
VST forward	5.31 (1.01)	5.42 (1.05)	5.38 (0.92)	5.42 (1.10)	0.22	0.641	0.001
VST backward	4.57 (1.13)	4.74 (1.24)	4.60 (1.14)	4.72 (1.18)	0.06	0.801	0.000
Fluency							
Categorical fluency	27.74 (5.87)	29.38 (5.92)	28.04 (5.74)	28.49 (6.07)	4.95	0.027	0.024
Language							
Boston Naming Test	11.69 (2.22)	12.22 (1.94)	11.51 (2.34)	11.69 (2.26)	5.02	0.026	0.025

a *M*(*SD*).

b Summation of total numbers (out of 45) of 5 times immediate recall of the word list.

c *p* Value from training group versus control group‐by‐time interaction with Bonferroni correction, SMCQ: Subjective Memory Complaints Questionnaire, SRFT: Simple Rey Figure Test, DST: Digit Span Test, VST: Visual Span Test.

**Figure 2:**
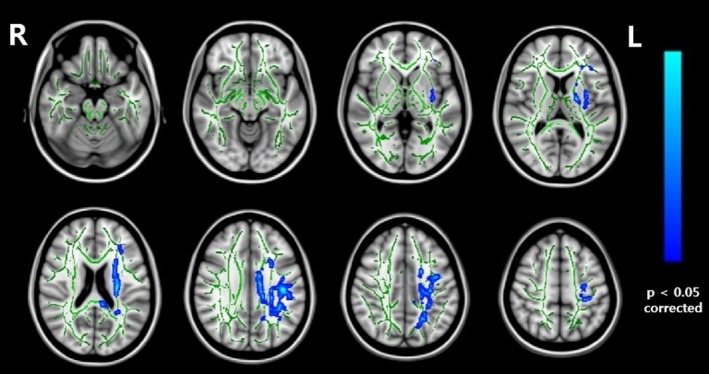
Tract‐based spatial statistics result of mean diffusivity (MD) changes between pre‐ and post‐training. The MD values were more decreased in the training than in the control group. These results are overlaid on the MNI 152 standard brain as skeleton image (green color, fractional anisotropy (FA) > 0.2). Images are presented with left as right, according to radiological convention, at statistical level of family‐wise error corrected *p* < 0.05

In the DTI analyses, the clusters showing a significant group‐time interaction on the MD encompassed five tracts: the left superior longitudinal fasciculus, left corona radiata (superior and posterior region), left external capsule, corpus callosum (body and splenium region), and the left posterior limb of the internal capsule. Those regions MD values were more decreased in the training than in the control group with FWE correction (*p* < 0.05) (Figure 2, Table 3, Appendix S1). There was no change in FA, RD, and AD with FWE correction (*p* < 0.05), while there was a trend of FA increase within the cluster in the training group.

**Table 3:** White matter regions showing significant group‐time interaction of mean diffusivity

MNI Coordinates			Cluster size	Location	*t*
*x*	*y*	*z*			
−40	−20	30	448	Superior longitudinal fasciculus L	3.58
−28	−18	19	381	Superior corona radiata L	3.60
−34	−8	−2	268	External capsule L	3.55
−9	−7	28	262	Body of corpus callosum	3.95
−20	−4	12	136	Posterior limb of internal capsule L	4.96
−25	−32	28	126	Posterior corona radiata L	3.71
−18	−36	31	109	Splenium of corpus callosum	4.48

**Figure 3:**
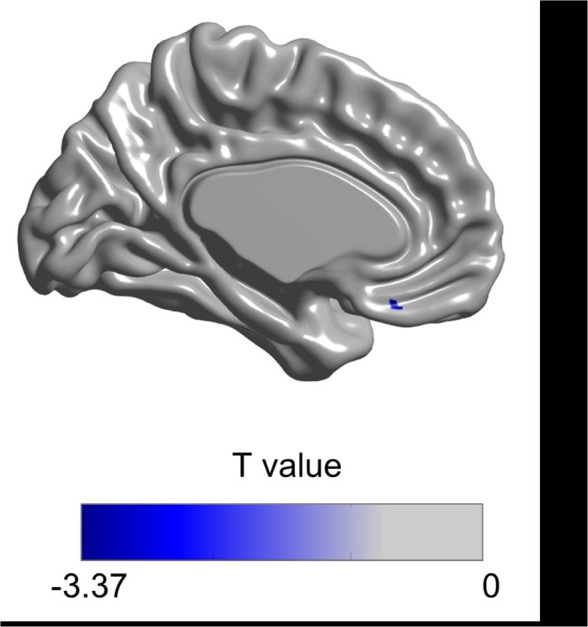
*T* value map of cortical thickness changes (post time point–pre‐time point) in the training group compared to the controls. The results revealed cortical changes in the training group compared to the controls in the left rectal gyrus (BA 11) at uncorrected *p* < 0.001. The color bar indicates *t* value range of −3.37 to 0. MNI coordinates at peak vertex of absolute t value is −3.41 mm (*x*), 29.05 mm (*y*), −20.73 mm (*z*)

In the cortical thickness analysis, the training group had more prefrontal cortical thickening of the left rectal gyrus (post‐pre‐evaluation) than the control group (uncorrected *p* < 0.001), but this effect did not persist after the FDR correction (Figure 3, Appendix S2).

## 4 DISCUSSION

We found that a multi‐strategic memory training based on the metamemory concept was effective in improving memory in healthy older adults with subjective memory impairment. Previous research on the metamemory abilities of older adults has reported that older adults express negative beliefs about their memory capability (Connor, Dunlosky, & Hertzog, 1997; Hultsch, Hertzog, & Dixon, 1987) and that they had poor strategies to control their memory abilities (Dunlosky & Connor, 1997). Therefore, a training regimen based on the metamemory concept may improve older adults’ memory ability by reducing anxiety and increasing memory strategy efficiency.

Scores in the delayed free recall of verbal memory, the categorical fluency, and the Boston Naming Test improved in the training group compared to the control group. The delayed recall ability is related to memory consolidation; therefore, it is challenging to train by simple memory training. Delayed recall ability in old age is very important as it is impaired during dementia, and it may be that by improving it the development of dementia will delay.

In addition, the intervention improved other cognitive abilities as well as memory. This may be related to improvement in self‐confidence and control over the memory process. However, although there has been no statistically significant difference, the reduction of the SMCQ scores in the two groups and the improvement of the memory functions in the training groups have been suitable to demonstrate the validity of the MMT and it is thought that further studies should be made in the future. Moreover, Recent studies have shown the impact of the metamemory process on the transfer of memory training to new domains, suggesting that multi‐strategic training with a metamemory approach may also facilitate encoding and retrieval through alternative metamemory processes including meta‐knowledge, meta‐monitoring, and meta‐judgment (Koriat, 2008; Koriat & Bjork, 2006).

However, other variables were not significant (MMSE, SMCQ, Visuospatial memory, and attention), but previous studies showed that metamemory task performance provides one indicator of self‐awareness of memory ability. Metamemory research is important because it allows an empirical approach to the broad construct “self‐awareness,” and can be extended as a framework to explore the processes and neural underpinnings of other cognitive, social, and sensory domains. Also, the previous study of metamemory experiments in neurological populations shows that there is a relationship between indices of frontal lobe function and metamemory accuracy and that there are many variables that affect metamemory performance such as the type of memory task, the format of memory task (recall or recognition), and type of meta‐judgment (Pannu & Kaszniak, 2005).

The MDs of the integrating regions such as the left superior longitudinal fasciculus and anterior corona radiata were more decreased after training, while their FAs are not. Prefrontal cortical thickening (i.e., rectal gyrus) tendency was also observed in the training group compared to the control group. The effect of cognitive training on the mean diffusivity suggests that DTI may be a useful marker of brain plasticity. MD change is more sensitive to white matter structural alterations than other types of changes. The cognitive training effect shown in fractional anisotropy was weaker than that shown in mean diffusivity. A decrease in mean diffusivity is a value reflecting myelination and axon density and is relatively constant in white matter, whereas an increase in fractional anisotropy reflects axonal integrity and myelination, but varies widely in the white matter. Therefore, small variations of myelination induced by training can only be detected by observing MD changes. Diffusional changes in Alzheimer's disease progression are also better shown in MD than in FA.

The left superior longitudinal fasciculus tract, which connects the frontal cortex with the parietal and temporal cortices, and anterior corona radiata tract, which is related to the prefrontal cortex, are changed after the MMT. This tract is the neuroanatomical foundation of various functions such as perception, emotion, and higher cognition. A previous longitudinal study demonstrated that multiple cognitive training induces differences in the superior longitudinal fasciculus tract compared to the control group (Cao et al., 2016). The medial prefrontal cortex was highly activated during the meta‐monitoring process (Do Lam et al., 2012), and reviews of brain research revealed that executive control and metacognition share the same brain region in the mid‐frontal area (Fernandez‐Duque, Baird, & Posner, 2000). Cognitive process speed (Turken et al., 2008), memory, and executive function (Bendlin et al., 2010) are related to integrity of the superior longitudinal fasciculus in healthy young adults compared to older adults. Memory training based on the metamemory concept may induce more prefrontal activation through the myelination of the anterior corona radiata tract. Furthermore, while the function of the rectal gyrus remains unclear, the region may related to higher cognitive functions such as planning or reasoning (Orrison, 2008). A larger increase of prefrontal cortical thickness in the training group compared to the control group indicates that multi‐strategic MMT helps to increase higher cognitive functions.

The limitation of this study is that there was no active control group receiving a separate type of training or educational intervention; however, it is useful to show that our intervention is superior to usual management. We did not measure anxiety and, therefore, have no data as to whether the decrease in anxiety was a mediator of the effect. We reduced bias by blinding the raters to the randomization status, but could not blind participants. We accounted for differences between centers using stratified randomization, but our power calculation and analyses did not account for clustering or for baseline cognition. Nonetheless, there was no difference between groups in cognition at baseline. Some participants in control group refused to enroll this study before agreement. Therefore, there was the failure to equalize the numbers of the two groups. We did not compare MRI changes in the non‐intervention groups with the intervention group. It would be useful for long‐term follow‐up to consider how long the changes lasted.

## 5 CONCLUSION

To the best of our knowledge, this is the first study that showed the effect of memory training with the metamemory concept using brain MRI. Our memory training may help older adults improve their memory ability and brain structures. Improved white matter integrity in the anterior and posterior cerebrum and increased cortical thickness of prefrontal regions, which are related to metacognition, possibly suggest that the effects of the MMT would be induced via the enhancement of cognitive control.
