# Supplementary figures and images for: Brain structural changes after multi‐strategic metamemory training in older adults with subjective memory complaints: A randomized controlled trial
Source: Brain Behav. 2019 Mar 27;9(5):e01278. doi: 10.1002/brb3.1278 (PMC6520300; doi:10.1002/brb3.1278)

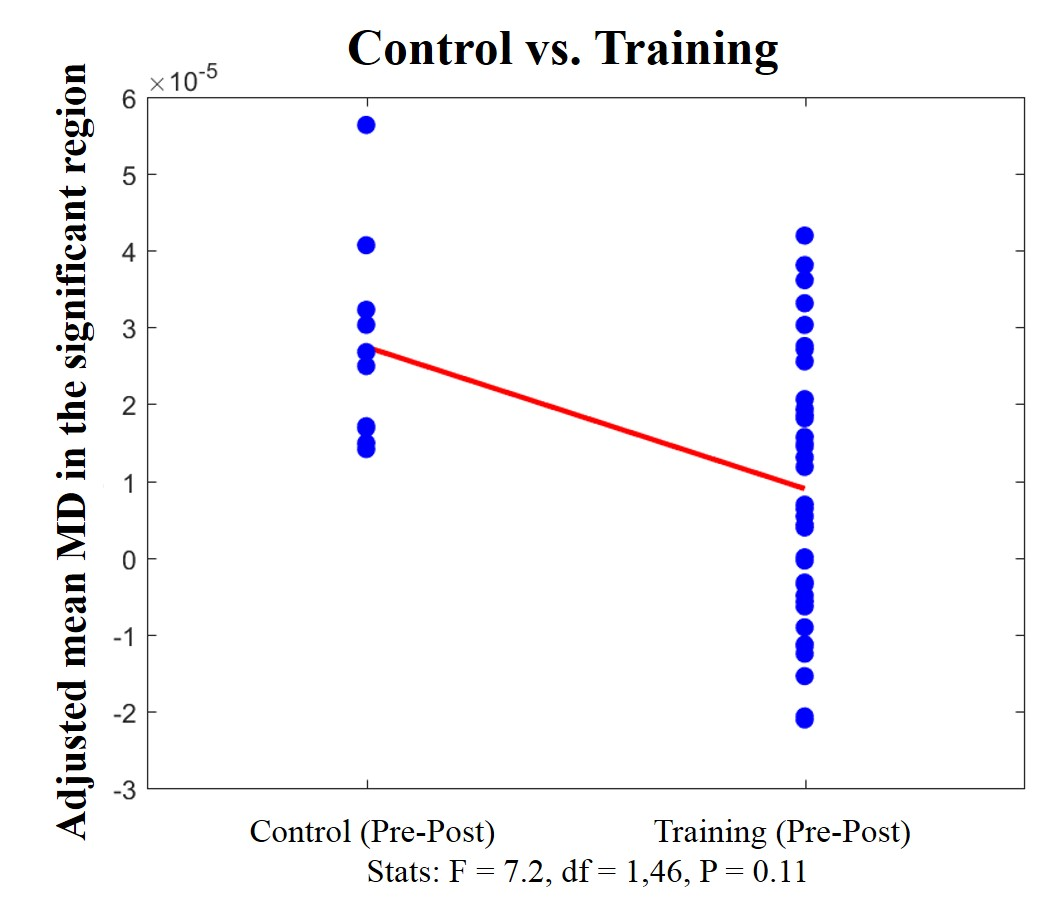

Supplement: Supplementary file 1 [file BRB3-9-e01278-s001.png]

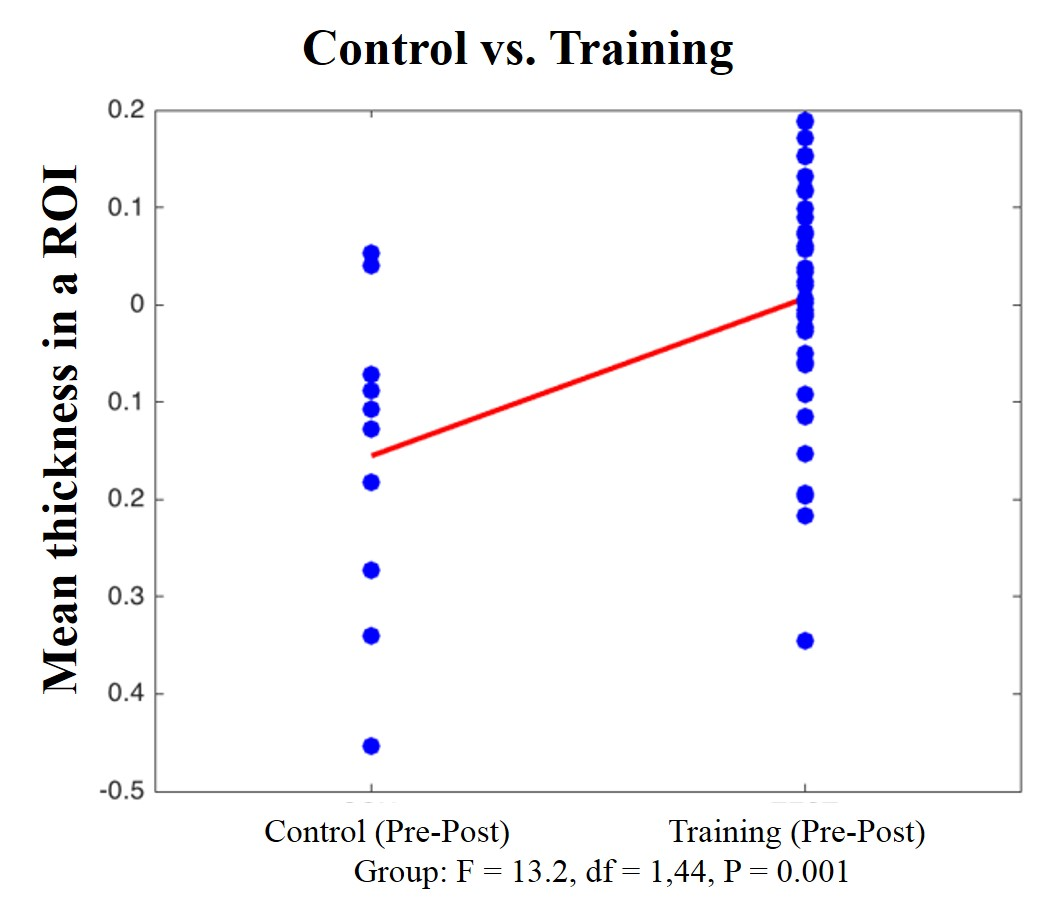

Supplement: Supplementary file 2 [file BRB3-9-e01278-s002.png]
